# Ankle Alignment Changes After High Tibial Osteotomy due to Valgus-Shaped Distal Tibia

**DOI:** 10.1177/19476035261465607

**Published:** 2026-08-12

**Authors:** Sem Dorst, Roel J.H. Custers, Nienke van Egmond, Ralph J.B. Sakkers, Harrie Weinans, Eva A. Bax

**Affiliations:** 1 Department of Orthopaedic Surgery, 8124 University Medical Centre Utrecht, Utrecht, The Netherlands; 2 Department Biomechanical Engineering, Faculty 3ME, TU Delft, Delft, The Netherlands

**Keywords:** ankle alignment, high tibial osteotomy, lower limb malalignment, radiographic analysis

## Abstract

**Purpose:**

Medial open-wedge high tibial osteotomy (MOW-HTO) is effective for correcting varus knee deformity but may unintentionally affect ankle joint alignment. This study investigated radiographic factors associated with postoperative ankle alignment changes following valgus-producing MOW-HTO.

**Methods:**

A retrospective analysis was conducted on a prospective cohort of 50 knees in 49 patients (mean age 46.3 ± 8.2 years). Patients were included if they underwent MOW-HTO between 2016 and 2024, were enrolled in the hospital knee registry, and had both pre-and postoperative whole-leg radiographs available. These weight-bearing long-leg radiographs were used to assess tibial bone deformity and ankle joint alignment. Postoperative ankle alignment was classified as deviating closer to neutral or further from neutral. Correlation analyses and within- and between-group comparisons were performed, with significance set at p < 0.05.

**Results:**

After MOW-HTO in varus-shaped knees, all alignment parameters shifted toward valgus. Tibial deformity predominantly originated from the distal tibia, with a medial center of rotation of angulation (CORA), reflecting an overall valgus-shaped tibia. Distal tibial valgus showed a strong correlation with talar orientation relative to the ground (ρ = 0.81, p < 0.001). Patients deviating further from neutral had nearly 5° more distal tibial valgus than those deviating closer to neutral (p < 0.001), and more frequently showed an overall valgus-shaped tibia (p < 0.01). Additionally, larger correction angles were moderately associated with greater valgus shift of the ankle (ρ = −0.44, p < 0.001).

**Conclusion:**

MOW-HTO results in a valgus shift of the talar orientation, particularly in patients with preoperative distal tibial valgus, an overall valgus-shaped tibia, and larger correction angles. Thorough preoperative assessment of tibial morphology is essential to better anticipate postoperative ankle alignment changes.

## 1. Introduction

Medial compartmental knee osteoarthritis (OA) is a common degenerative joint disease frequently associated with varus lower limb malalignment. Medial open-wedge high tibial osteotomy (MOW-HTO) is an effective joint-preserving treatment in which the mechanical medial proximal tibial angle (mMPTA)—the angle between the tibial plateau and mechanical axis of the tibia^1^ —is corrected, thereby shifting load from the affected medial to the unaffected lateral compartment.^2^ Favourable outcomes have been reported, with high patient satisfaction and improvements in pain and function.^3-10^ However, MOW-HTO may also induce compensatory reorientation of the ankle joint,^11-15^ which can cause or increase ankle joint malalignment with concomitant ankle joint pain.^12^ These changes may contribute to the development of ankle OA, ultimately leading to functional impairment and reduced quality of life.^16^

**Figure 1.**
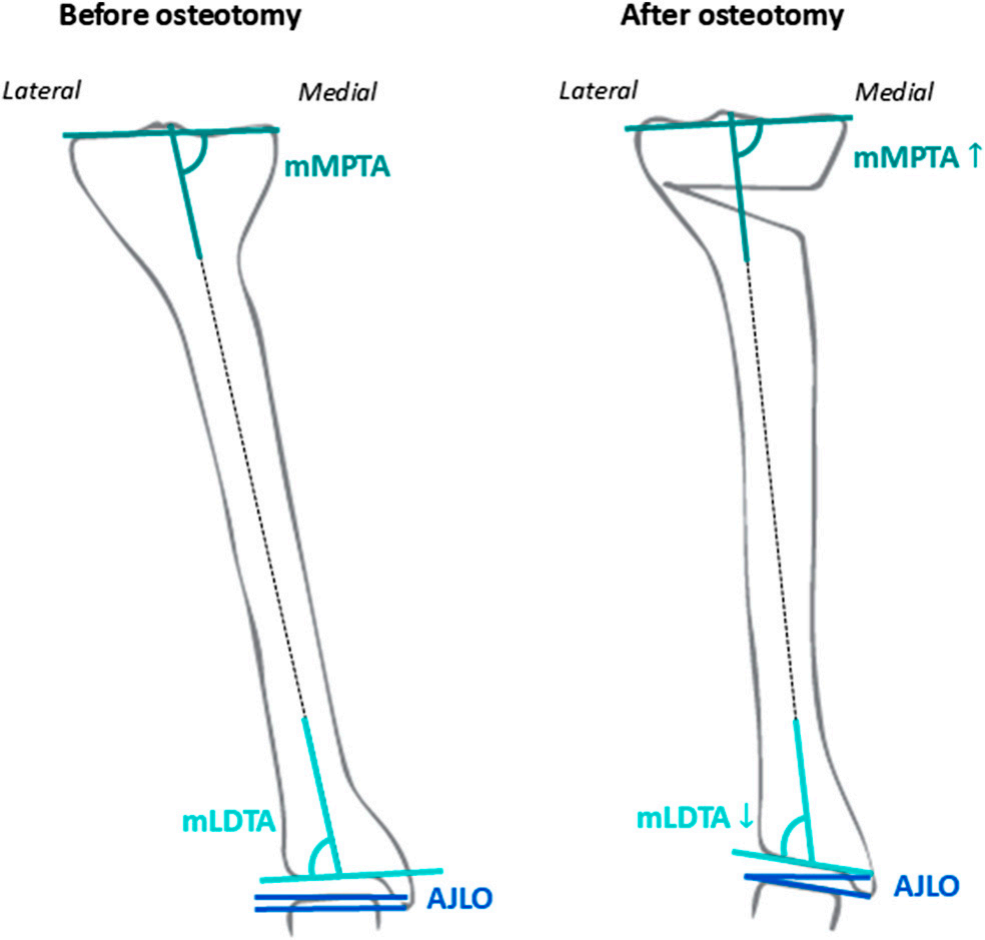
Schematic representation of angular changes in lower limb alignment following MOW-HTO. The procedure results in an increase of the mMPTA, indicating a shift toward valgus alignment at the knee. Correspondingly, the mLDTA also tends to increase toward valgus. This valgus correction is further reflected at the ankle, where the AJLO typically changes from a neutral toward a more valgus alignment

In recent years, ankle joint alignment has gained increasing recognition as an important consideration in the context of MOW-HTO, although the factors contributing to postoperative ankle joint malalignment remain poorly understood.^13^ A recent systematic review highlighted the need for studies that include both pre- and postoperative radiographic measurements to better identify risk factors for altered ankle joint alignment.^11^ From a biomechanical perspective, several parameters are involved in postoperative ankle joint orientation. Following MOW-HTO, the mMPTA increases, shifting more toward valgus (Figure 1). Concurrently, the mechanical lateral distal tibial angle (mLDTA)—the angle between the mechanical axis of the tibia and the distal joint line^1^ —also typically shifts toward valgus (Figure 1). This valgus tendency is further reflected at the ankle joint, where the Ankle Joint Line Obliquity (AJLO)—defined as the angle between the talar dome and the horizontal plane^17^ —generally tilts further into valgus (Figure 1). The center of rotation of angulation (CORA), defined as the intersection of the mechanical axes in a deformed bone,^1^ indicates whether the overall tibia exhibits a deformity, as it reflects the relationship between the proximal and distal tibial joint lines. When the CORA is located more distally, the deformity primarily originates from the mLDTA, whereas a more proximal CORA indicates that the deformity arises from the mMPTA. In addition, the mediolateral position of the CORA provides insight into the overall alignment of the tibia: a medially located CORA is associated with an overall valgus alignment, while a laterally located CORA corresponds to an overall varus alignment. Consequently, the CORA influences changes in the AJLO; a medial CORA leads to alignment further from neutral (more to valgus), whereas a lateral CORA results in alignment closer to neutral (more to varus). These biomechanical relationships form the basis of the hypotheses of this study.

Taken together, these observations suggest that the mLDTA, CORA location, and the degree of mMPTA correction may collectively influence postoperative AJLO, as a MOW-HTO inherently creates a more valgus alignment. Consequently, if the mLDTA or CORA already exhibit a valgus alignment preoperatively, the postoperative valgus alignment of the ankle joint may become further pronounced. To address this gap, the present study had two objectives. First, to determine the alignment of the tibiotalar in tibiae considered suitable for a valgus-producing MOW-HTO. Second, to investigate the association between the mLDTA, CORA location, and the degree of mMPTA correction with postoperative ankle joint alignment in patients undergoing MOW-HTO. A better understanding of these parameters may improve preoperative planning and understanding of postoperative radiographic ankle alignment changes.

## 2. Methods

### 2.1 Study Population

This study was a retrospective analysis of a prospective cohort of patients treated in a university medical center in the Netherlands. All patients who underwent MOW-HTO between 2016 and 2024 and were enrolled in the hospital’s Knee Registry were considered for inclusion. Patients who underwent a distal femoral osteotomy or a double-level osteotomy, or who lacked postoperative weight-bearing long-leg radiographs (WLRs) (N = 9) were excluded from the population. No patients were excluded due to pre-existing ankle problems. Demographic and clinical characteristics, including age, gender, body mass index (BMI), operated side, and degrees of correction were recorded. This study does not fall under the scope of the Dutch Medical Research Involving Human Subjects Act (WMO). It therefore does not require approval from an accredited medical ethics committee in the Netherlands. However, an independent quality check has been carried out to ensure compliance with legislation and regulations (regarding informed consent procedure, data management, privacy aspects and legal aspects).

### 2.2 Radiographic Assessment

Pre- and postoperative WLRs were used to assess several coronal-plane radiological parameters, including the Hip-Knee-Ankle Angle (HKAA), mMPTA, Joint Line Convergence Angle (JLCA), mLDTA, CORA, and AJLO. The WLRs were acquired according to a previously published and standardized protocol,^18^ which has demonstrated test–retest variability of <1° for knee geometry parameters. Previous studies showed good to excellent inter- and intra-observer reliability for these measures.^13,14,18,19^

**Figure 2.**
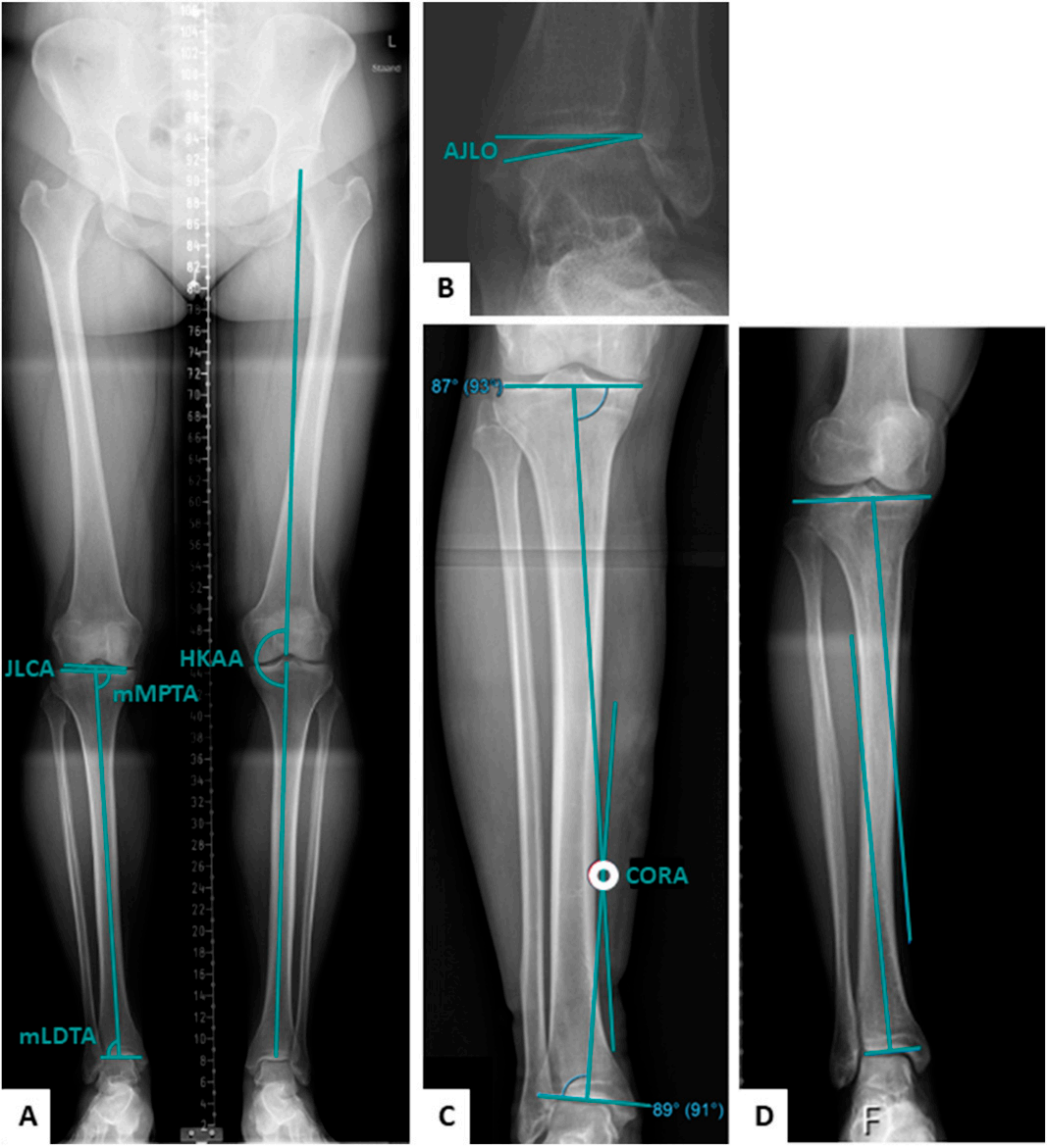
Radiographic measurements on whole leg radiographs. (A) Hip-Knee-Ankle Angle (HKAA), Medial Proximal Tibial Angle (mMPTA), Joint Line Convergence Angle (JLCA), and mechanical Lateral Distal Tibial Angle (mLDTA). (B) Valgus Ankle Joint Line Obliquity (AJLO) C) Center of Rotation and Angulation (CORA), located medially. (D) CORA absent as the lines of the mechanical axes are parallel

The HKAA (Figure 2A) was defined as the angle between the mechanical axis of the femur and the tibia, while the mMPTA (Figure 2A) represented the angle between the mechanical axis of the tibia and the proximal tibial plateau. The JLCA (Figure 2A) was defined as the angle between the distal femoral and proximal tibial joint lines. The mLDTA (Figure 2A) indicated the angle between the mechanical axis of the tibia and the distal joint line of the tibia.^1^ Normal values for these angles are presented in Table 1.

**Table 1.** The Alignment Classification for Normal, Varus and Valgus Alignment

​	Valgus	Neutral	Varus
mMPTA	> 90°	|85° - 90°|	< 85°
JLCA	< 0°	|0° - 2°|	> 2°
mLDTA	< 86°	|86° - 92°|	> 92°

mMPTA, mechanical medial proximal tibial angle; JLCA, Joint Line Convergence Angle; mLDTA, mechanical lateral distal tibial angle.

The AJLO (Figure 2B) was defined as the angle between the talar dome and the horizontal plane.^17^ The change in AJLO (ΔAJLO) was calculated as the postoperative value minus the preoperative value, with negative values indicating a shift toward valgus and positive values indicating a shift toward varus.

Patients were categorized into three groups based on Δ AJLO: those whose ankle joint alignment deviated closer to neutral (N = 13), those with unchanged alignment (change between −1° and +1°, N = 9), and those whose alignment deviated further from neutral (N = 28).

The CORA (Figure 2C) was defined as the intersection of the proximal and distal mechanical axes (PMA and DMA) of the tibia. The PMA, representing the mechanical axis of the proximal tibia, was drawn through the center of the proximal tibia at a medial angle of 87° relative to the proximal tibial joint line.^1^ The DMA, representing the mechanical axis of the distal tibia, was drawn through the center of the distal tibia at a lateral angle of 89° relative to the distal tibial joint line.^1^ Patients were categorized into two groups based on the presence or absence of a CORA. Furthermore, patients with a CORA were subcategorized according to its location—medial, lateral, or within the tibial shaft—as a medially located CORA is associated with an overall valgus alignment, whereas a lateral located CORA corresponds to an overall varus alignment. Moreover, patients were further classified based on the proximal or distal position of the CORA, with a more proximally located CORA indicating that the deformity arises from the mMPTA, whereas a more distally located CORA suggests that the deformity primarily originates from the mLDTA.

All measurements were performed by a single observer using standardized methods within the Picture Archiving and Communication System (PACS, Sectra AB, Linköping, Sweden). Measurements were recorded and subsequently verified by a second observer. For five sets of measurements, the second observer performed independent measurements, which showed good reliability with the first observer. All other measurements were recorded by the first observer and checked by the second observer.

### 2.3 Potential Risk Factors for Ankle Joint Malalignment

Potential risk factors for postoperative ankle joint malalignment after MOW-HTO were evaluated using radiographic and geometric parameters, with the AJLO as the outcome measure. First, the mLDTA was analysed. Second, the presence and location of the CORA were assessed and classified as medial (indicative of valgus alignment), lateral (indicative of varus alignment), or within the tibial shaft. Third, the position of the CORA was expressed as a percentage of the tibial length, with 100% corresponding to the proximal tibia and 0% to the distal tibia. Finally, the influence of the correction angle was examined.

### 2.4 Statistical Analysis

Statistical analyses were performed using IBM SPSS Statistics for Windows, version 30 (IBM Corp., Armonk, NY, USA). Continuous variables are presented as mean ± standard deviations (SD) or median (interquartile range (IQR)), depending on normality, which was assessed using the Shapiro-Wilk test. Paired t-tests and Wilcoxon Signed Ranks tests were used for within-group comparisons, while independent t-tests and Mann-Whitney U tests were applied for between-group comparisons. Spearman’s rho was used to assess correlations, and categorical variables were compared using the Chi-squared test. P-values< 0.05 were considered statistically significant.

A sample size analysis was performed using G*Power 3.1^20^ (Heinrich-Heine-Universität, Düsseldorf, Germany). Based on an observed difference in mLDTA, the estimated effect size was -0.91, corresponding to a required sample size of sixteen patients to detect a statistically significant difference with 90% power (α = 0.05).

## 3. Results

### 3.1 Study Population

A total of 50 knees from 49 patients were included, of whom 58% were male. The mean age was 46.3 ± 8.2 years, and the mean BMI was 26.8 ± 3.2 kg/m^2^. The mean interval to postoperative weight-bearing radiographs was 3.7 ± 2.5 months.

Postoperatively, the HKAA, mMPTA, mLDTA, and AJLO all shifted significantly toward valgus alignment compared with preoperative values (Table 2). Specifically, for the AJLO, two ankle joints (4.0%) remained in varus, one ankle joint (2.0%) showed no change, and 47 ankle joints (94.0%) shifted to valgus following MOW-HTO.

**Table 2.** Preoperative and Postoperative Radiographic Coronal Alignment Parameters Following MOW-HTO

​	Preoperative	Postoperative	P-value
HKAA (mean ± SD)	184.3 ± 2.5°	178.5 ± 2.5°	< 0.001
mMPTA (mean ± SD)	86.2 ± 2.2°	91.2 ± 3.1°	< 0.001
JLCA (median (IQR))	2.0° (2.0°)	2.0° (2.0°)	0.13
mLDTA (median (IQR))	86.0° (6.0°)	85.0° (4.0°)	< 0.01
AJLO (median (IQR))	1.0° (6.0°)	−4.0° (4.0°)	< 0.001

HKAA, Hip-Knee-Ankle Angle; SD, Standard deviations; mMPTA, Mechanical medial proximal tibial angle; IQR, Interquartile range; JLCA, Joint Line Convergence Angle; mLDTA, Mechanical lateral distal tibial angle; AJLO, Ankle joint line obliquity.

### 3.2 Effect of mLDTA on Ankle Joint Alignment

**Figure 3.**
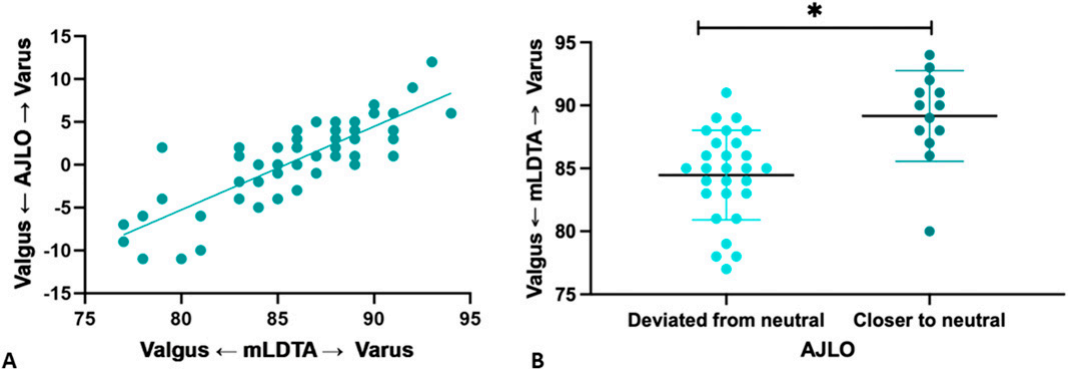
Relationship between preoperative medial lateral distal tibial angle (mLDTA) and ankle joint line obliquity (AJLO). (A) Scatter plot showing a strong positive correlation between preoperative mLDTA and preoperative AJLO. (B) Swarm plot illustrating the preoperative mLDTA in patients whose AJLO deviated further from neutral and those whose AJLO deviated closer to neutral, showing significantly more valgus mLDTA values in patients whose alignment deviated further from neutral

Preoperatively, 23 (46.0%) tibiae demonstrated a valgus mLDTA (mean 82.1 ± 2.9°), 2 (4.0%) a varus mLDTA, and 25 (50.0%) a neutral mLDTA (mean 88.5 ± 1.9°). Preoperative mLDTA was strongly and positively correlated with preoperative AJLO (ρ = 0.81, P < 0.001, 95% CI 0.68 to 0.89; Figure 3A). A more valgus mLDTA was associated with a more valgus orientation of the AJLO.

Analysis of AJLO changes after MOW-HTO revealed that patients whose ankle joint alignment deviated further from neutral had a statistically significantly lower preoperative mLDTA (84.5 ± 3.6°) compared with those whose alignment deviated closer to neutral (89.2 ± 3.6°; P < 0.001), indicating more preoperative valgus alignment in the group that deviated further from neutral (Figure 3B).

### 3.3 Effect of CORA on Ankle Joint Alignment

A CORA was present in 33 knees (66.0%, Figure 2C) and absent in 17 knees (34.0%, Figure 2D). Among knees with a CORA, it was located medially (indicative of valgus alignment) in 15 knees (45.5%, Figure 2C), laterally (indicative of varus alignment) in 8 knees (24.2%), and within the tibial shaft in 10 knees (30.3%).

**Figure 4.**
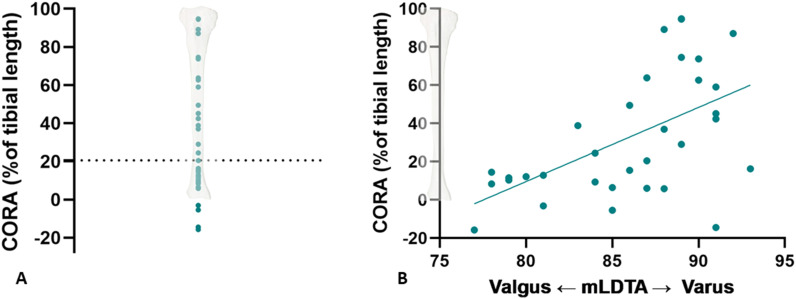
CORA location expressed as a percentage of the tibial length. (A) Distribution of the CORA location, with the dotted line indicating the median value at 20.5%. (B) Scatter plot showing the relationship between CORA location (% of tibial length) and the preoperative mechanical lateral distal tibial angle (mLDTA). (A) more valgus mLDTA was associated with a more distally located CORA

The median location of the CORA as a percentage of the tibial length was 20.5% (IQR 52.0), with 100% representing the proximal tibia and 0% the distal tibia (Figure 4A). This indicates that, on average, the CORA was located in the distal fifth of the tibia. There was a statistically significant moderate correlation between the location of the CORA (as a percentage of the tibial length) and the preoperative mLDTA (ρ = -0.55, P < 0.001, 95% CI -0.76 to -0.25). A preoperative valgus mLDTA was significantly associated with a more distally located CORA (Figure 4B).

**Figure 5.**
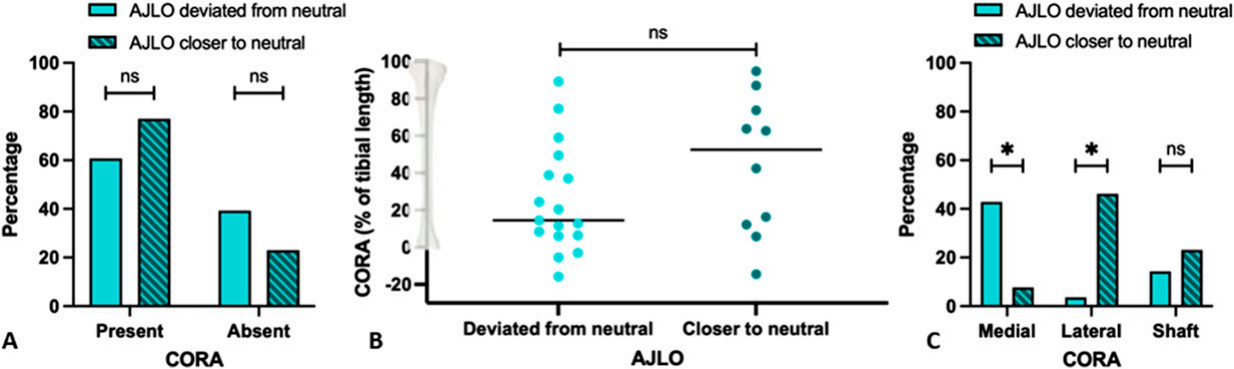
Comparison of CORA characteristics between patients whose AJLO deviated further from neutral and those whose AJLO deviated closer to neutral. (A) Proportion of tibiae with and without a visible CORA, showing no significant difference between groups. (B) Swarm plot of CORA location as a percentage of tibial length, showing no significant difference between groups; horizontal lines indicate medians. (C) Distribution of CORA location categorized as medial, lateral, or within the tibial shaft, with significantly more medial CORAs in patients whose alignment deviated further from neutral and more lateral CORAs in those whose alignment deviated closer to neutral

Analysis of CORA characteristics in relation to AJLO changes showed no statistically significant differences in the presence or absence of a CORA between the groups whose ankle alignment deviated closer to neutral (77% present, 23% absent) and those whose alignment deviated further from neutral (60.7% present, 39.3% absent) (P = 0.63) (Figure 5A). Similarly, the location of the CORA as a percentage of the tibial length did not differ significantly between the two groups (52.5% vs. 14.4%, P = 0.22) (Figure 5B).

In contrast, CORA location differed significantly between groups. Medial CORAs (valgus tibia) were more frequent in patients whose ankle joint alignment deviated further from neutral (42.9%) compared with those whose alignment deviated closer to neutral (7.7%) (P < 0.01), whereas lateral CORAs (varus tibia) were more common in patients whose alignment deviated closer to neutral (46.2%) than in those whose alignment deviated further from neutral (3.6%) (P < 0.01) (Figure 5C). The mean ΔAJLO was 2.7 ± 4.2° toward valgus for medial CORAs and 5.5 ± 4.4° toward valgus for lateral CORAs; this difference did not reach statistical significance (P = 0.15).

### 3.4 Effect of mMPTA Correction on Ankle Joint Alignment

**Figure 6.**
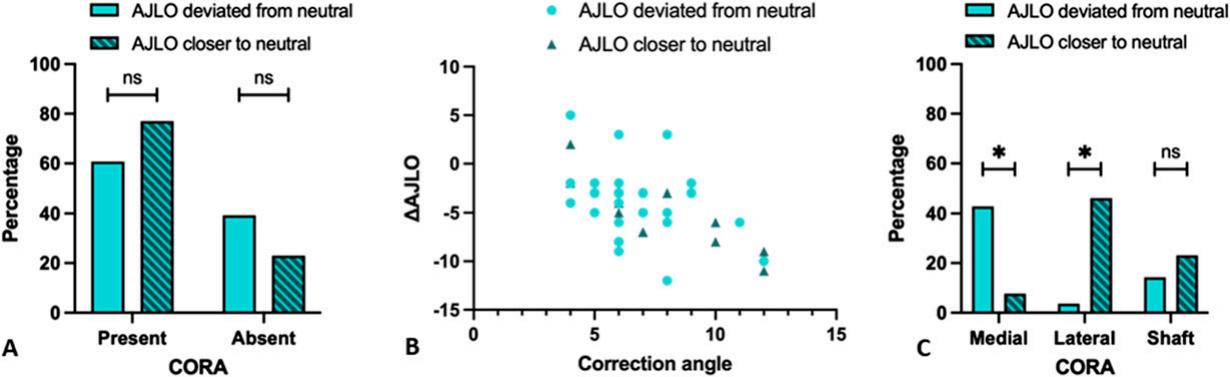
Relationship between the correction angle and change in ankle joint line orientation (ΔAJLO). Data are shown according to AJLO outcome groups: deviated from neutral and closer to neutral

The median correction angle was 7.0° (2.0°). A moderate negative correlation was observed between the correction angle and ΔAJLO (ρ = −0.44, P < 0.001, 95% CI -0.65 to -0.18; Figure 6), indicating that larger correction angles were associated with a greater shift toward valgus AJLO. However, no statistically significant difference in correction angle was found between ankles that deviated closer to neutral (7.0° (4.0°)) and those that deviated further from neutral (6.0° (3.0°); P = 0.20).

## 4. Discussion

In this study, we aimed to evaluate the alignment of the tibiotalar joint in tibiae with varus knees considered suitable for valgus-producing MOW-HTO. Preoperatively, 46.0% of tibiae demonstrated a valgus mLDTA, 50.0% a neutral mLDTA, and 4.0% a varus mLDTA. In most cases, the CORA was located medially, indicating an overall valgus tibial alignment. The CORA was located in the distal fifth of the tibia, suggesting that the deformity primarily originated from the distal tibial alignment, as reflected by the mLDTA. These findings suggest that, in these patients indicated for valgus-producing MOW-HTO, the tibial deformity primarily originates from the distal tibia (mLDTA) rather than from the proximal tibia (mMPTA). ^20^

A secondary aim of this study was to investigate factors contributing to postoperative changes in AJLO in patients undergoing valgus-producing MOW-HTO, with specific focus on the mLDTA, the CORA, and the degree of mMPTA correction. Preoperative mLDTA was strongly correlated with preoperative AJLO, suggesting that the distal tibial alignment strongly influences the orientation of the ankle joint line preoperatively. This subgroup of patients may potentially benefit from conservative treatment options such as the use of inlays, which warrants further investigation. Patients whose ankle alignment deviated further from neutral after MOW-HTO had significantly lower (more valgus) preoperative mLDTA compared with those whose alignment deviated closer to neutral, suggesting that preexisting valgus alignment of the distal tibia predisposes to further deviation from neutral in postoperative ankle joint alignment. A moderate correlation was found between the preoperative mLDTA and the CORA location (as a percentage of the tibial length), with a more valgus mLDTA being associated with a more distally located CORA. Moreover, patients whose postoperative ankle joint alignment deviated further from neutral exhibited significantly more medially located CORAs—consistent with valgus alignment—than those whose alignment moved closer to neutral. In other words, when the tibial deformity originates medially (valgus-type CORA), the ankle joint tends to shift further into valgus after correction. Although the CORA tended to be located more distally in patients whose ankle alignment deviated further from neutral compared with those whose alignment deviated closer to neutral, this difference did not reach statistical significance. Finally, a moderate negative correlation was observed between the correction angle and ΔAJLO, indicating that larger correction angles were associated with a shift of the ankle joint line toward valgus. These findings highlight the importance of assessing distal tibial alignment when evaluating candidates for valgus-producing MOW-HTO in order to minimize potential postoperative radiographic changes in ankle alignment, a concept that is also supported by research in total knee arthroplasty showing that knee deformities can influence postoperative ankle alignment and function.^21^

Furthermore, as expected after a MOW-HTO, the HKAA, mMPTA, and mLDTA all shifted toward valgus. This confirms the intended correction effect of the procedure for the HKAA and mMPTA, albeit not favorable for the mLDTA. The valgus shift in mLDTA is in line with biomechanical principles; however, the literature on this topic is not entirely consistent. While some studies report a significant change in mLDTA after osteotomy,^13^ others have found no statistically significant difference.^22^ Interestingly, the CORA could be identified in 66% of cases, while it was absent in 34%. To our knowledge, no previous studies have reported on the prevalence of the CORA in adult patients with varus malalignment. The absence of a CORA may be explained by either a straight tibia without evident malalignment, or by a primarily translational deformity characterized by an S-shaped tibial contour (Figure 2D), leading to non-intersecting mechanical axes.^1^

Postoperative changes in ankle joint alignment following MOW-HTO seem to have important clinical implications, although these clinical implications were not demonstrated in this study. In literature, new-onset unexplained ankle symptoms have been reported in 14–20% of cases, with a mean onset of symptoms around 20 months after surgery.^23,24^ Ankle joints that are less parallel to the ground postoperatively are associated with significantly more ankle pain compared to those with more neutral alignment at a mean follow-up of over two years.^12^ Moreover, patients with preexisting ankle OA experience a significant worsening of pain after MOW-HTO,^23-25^ and ankle joint alignment deviated further from neutral has been associated to increased patient-reported pain and may contribute to the development or progression of ankle OA, resulting in functional impairment and reduced quality of life.^12,16,26^ Another study demonstrated that ankle malalignment was associated with increased contact pressure, which elevates the risk of cartilage thinning, degeneration, and the development of ankle OA.^27^ Although some studies found no association between postoperative ankle joint alignment and short-term clinical outcome scores at one year,^28^ the long-term data suggest that malalignment can have meaningful consequences. Taken together, these findings indicate that patients with a valgus alignment of the distal tibia may be particularly susceptible to postoperative radiographic changes in ankle alignment, highlighting the need for careful consideration of whether these patients truly benefit from proximal tibial osteotomies.

At birth, the lower limbs show a varus alignment that gradually shifts to valgus, peaking around 3–5 years of age, and stabilizing by age seven to a physiological pattern similar to adults, typically with a slight varus HKAA and mMPTA.^29,30^ In our cohort, the HKAA shows varus alignment, whereas the mLDTA appears neutral or valgus. While 64–75% of patients with knee OA^31-33^ and 13–25% of individuals without knee OA^34-37^ have a varus knee alignment, comparable data for mLDTA are scarce. Limited evidence suggests that men tend to have a more valgus mLDTA than women.^38^ These findings raise questions about the underlying deformity patterns observed in patients undergoing corrective osteotomy. A MOW-HTO is typically performed to correct a varus deformity of the knee by creating a valgus-producing osteotomy in the proximal tibia. However, in our cohort, 15 patients demonstrated a valgus tibial alignment, as indicated by a medially located CORA. This implies that knee surgeons frequently perform a valgus-producing osteotomy on a tibia that is already in valgus when considering the CORA location. Among these, 12 patients showed a postoperative deviation of ankle alignment further from neutral following MOW-HTO. Future studies should investigate whether modified surgical strategies, such as a double-level osteotomy involving both proximal and distal tibial corrections, could prevent postoperative deterioration of ankle joint alignment and potentially improve clinical outcomes. A distal tibial osteotomy alone primarily alters alignment within the distal tibia and therefore has no direct effect on the HKAA or on the mechanical load distribution across the knee joint. Combining proximal and distal corrections may, however, allow for a more balanced restoration of both knee and ankle alignment.

In recent years, several studies have reported significant alterations in coronal ankle joint alignment following MOW-HTO.^12,13,39^ Lower preoperative mLDTA values have been associated with a more valgus ankle joint orientation,^11-13,39^ and the degree of coronal correction has been correlated with the magnitude of AJLO change.^12,24^ However, no studies have yet examined the influence of CORA location on postoperative ankle joint alignment. Building on this, the present study combines a biomechanical rationale with clinical validation, not only theorizing the effect of mLDTA, CORA location, and correction magnitude on ankle joint alignment but also confirming these relationships using radiological patient data. This approach provides new insights into preoperative radiological parameters that are associated with postoperative ankle joint orientation and may guide more individualized surgical planning. The sample size was substantial, and all patients underwent standardized postoperative whole-leg radiographs at four months, ensuring consistent assessment of coronal alignment.

Despite the strengths, several limitations should be considered. First, for ankle joint alignment, only AJLO was assessed in this study, whereas other parameters, such as talar tilt, were not included. Second, clinical outcome data were limited, restricting the ability to directly correlate radiographic changes with patient-reported outcomes, especially the long-term clinical effect on the ankle joint. Third, all radiographic measurements were performed by a single observer and subsequently checked by a second observer, providing internal verification, although measurements were not performed independently. However, previous studies have demonstrated high interobserver reliability for these lower limb alignment parameters.^18,40^ Fourth, several statements in this study imply mechanistic conclusions; however, important potential confounding factors—such as pelvic tilt, ankle osteoarthritis severity, subtalar joint mobility, and foot progression angle—were not measured and may have influenced ankle alignment. Nevertheless, the measurement of mLDTA and CORA in patients with knee varus malalignment undergoing MOW-HTO provides valuable insight into which individuals may be at risk of postoperative deterioration in ankle joint alignment. These findings raise the possibility that patients with a valgus mLDTA could benefit from modified surgical strategies, such as additional distal tibial corrections, to prevent postoperative malalignment; however, this hypothesis requires further investigation in future studies. Furthermore, future studies could implement weightbearing cone-beam computed tomography to more accurately assess subtalar joint alignment, overcoming limitations of superimposition on conventional radiographs.^41^

## 5. Conclusion

In conclusion, this study aimed to determine the tibiotalar alignment in tibiae considered suitable for a valgus-producing MOW-HTO and to investigate the relationship between the mLDTA, CORA location, and the degree of mMPTA correction with postoperative ankle joint alignment. The findings indicate that the CORA was most frequently positioned medially within the distal tibia, suggesting an overall valgus configuration of the tibia. This shape is mostly the consequence of a valgus mLDTA and is associated with postoperative deviation of ankle joint alignment further from neutral, resulting in an even more valgus shape. Moreover, larger mMPTA correction angles correlated with greater postoperative changes in ankle joint alignment. These findings emphasize the importance of a comprehensive preoperative evaluation of tibial morphology to better anticipate postoperative talar changes. Future osteotomy studies should focus on ankle pain and the development of ankle OA following knee osteotomy, and investigate whether modified surgical strategies can prevent postoperative ankle malalignment.

## Appendix

### List of Abbreviations

AJLO: Ankle joint line obliquity
BMI: Body mass index
CORA: Center of rotation of angulation
DMA: Distal mechanical axes
HKAA: Hip-Knee-Ankle Angle
IQR: Interquartile range
JLCA: Joint Line Convergence Angle
mLDTA: Mechanical lateral distal tibial angle
mMPTA: Mechanical medial proximal tibial angle
MOW-HTO: Medial open-wedge high tibial osteotomy
OA: Osteoarthritis
PACS: Picture Archiving and Communication System
PMA: Proximal mechanical axes
SD: Standard deviations
WLRs: Weight-bearing long-leg radiographs.
